# Circular Single-Stranded DNA Virus (*Microviridae*: *Gokushovirinae*: *Jodiemicrovirus*) Associated with the Pathobiome of the Flat-Back Mud Crab, *Eurypanopeus depressus*

**DOI:** 10.1128/MRA.01026-19

**Published:** 2019-11-21

**Authors:** Jamie Bojko, Krista A. McCoy, Donald C. Behringer, April M. H. Blakeslee

**Affiliations:** a Emerging Pathogens Institute, University of Florida, Gainesville, Florida, USA; b Biology Department, East Carolina University, Greenville, North Carolina, USA; KU Leuven

## Abstract

A single-stranded DNA (ssDNA) virus is presented from a metagenomic data set derived from *Alphaproteobacteria*-infected hepatopancreatic tissues of the crab Eurypanopeus depressus. The circular virus genome (4,768 bp) encodes 14 hypothetical proteins, some similar to other bacteriophages (*Microviridae*). Based on its relatedness to other *Microviridae*, this virus represents a member of a novel genus.

## ANNOUNCEMENT

*Microviridae* is a viral family with two subfamilies and 6 genera (1). It contains viruses that infect prokaryotes exclusively. Metagenomic techniques have unearthed the diversity of this family through evaluations of environmental, culture stock, and animal specimens (1–3). Microviruses have not been identified from *Rickettsiales* endosymbionts of Crustacea. Other bacteriophages isolated from crustacean microbiomes/pathobiomes show potential for phage therapy, avoiding the overuse of antibiotics in aquaculture (4). Few models exist to easily test this scenario in the laboratory.

**FIG 1:**
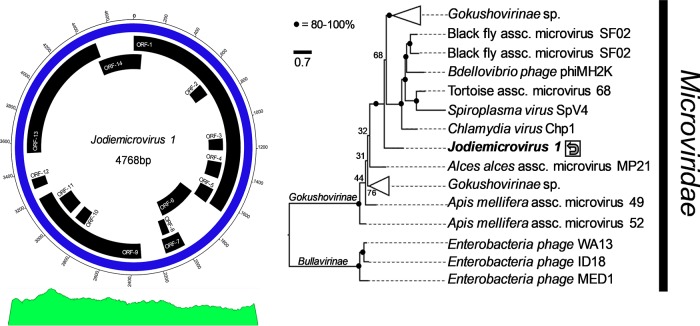
Circular genome of *Jodiemicrovirus 1*, consisting of 4,768 bp, and phylogenetic comparison to other *Microviridae* using the capsid protein (ORF-1). The genome contains 14 hypothetical open reading frames. The chart at the bottom identifies the read coverage across the circular genome, representing 934,456 reads mapped to the genome, providing >1,000× coverage using CLC Genomics Workbench. The phylogenetic comparison included the MAFFT-aligned (11) capsid protein (716 positions) from multiple *Microviridae*. The maximum likelihood tree was inferred from 36 *Microviridae* spp. and was developed with the LG+F+G4 evolutionary model and 1,000 bootstraps in IQ-Tree (10). The final consensus tree (shown) had a log likelihood of −28,362.192 and scale of 0.7 units. The accession numbers used were AXL15123, AXQ65957, QCS36953, AXH77578, AXL15643, AZL82997, AZL82921, AXL14929, YP_009218802, AYQ58216, AXL14945, AZL82910, AZL82729, AZL83022, AZL83017, QCS36934, QCS36961, QCS37361, AZL82956, AZL82992, AZL82871, YP_009551424, AZL82946, QCQ84972, AZL82926, QCS37201, QCQ84913, AZL82717, AZL82837, YP_512416, YP_512796, AII27899, NP_073538, NP_044312, and NP_598320.

We obtained DNA using a Zymo kit (D4070) on homogenized hepatopancreatic tissues of Eurypanopeus depressus (*n* = 1), a panopeid crab from meso- and euryhaline locations across the Gulf of Mexico and Atlantic North America. The specimen was collected from a euryhaline site in North Carolina (Hoop Pole Creek, Atlantic Beach) in December 2018. A total of 1 μg of DNA was used to prepare a NEBNext Ultra DNA library for Illumina HiSeq (10×) sequencing (NEB, USA) with a PE150 cartridge. This resulted in 11 million reads (50 to 150 bp) that were assembled using SPAdes v.3.13.0 (using default parameters and k-mer lengths of 21, 33, 55, 77, 99, and 127) (5) from trimmed reads using Trimmomatic (LEADING:3 TRAILING:3 SLIDINGWINDOW:4:15 MINLEN:36) (6). This resulted in 523,047 contigs (>500 bp) (*N*_50_, 2,133; *N*_75_, 1,340; *L*_50_, 100,989; *L*_75_, 211,827). The genome (4,768 bp) of a *Microviridae* sp. was identified based on high coverage (>1,000×), with a GC content of 33% and 14 hypothetical open reading frames (ORFs) (Fig. 1 and Table 1). The genome was annotated using ExPASy (standard genetic code) (7) and GeneMarkS (virus) (8). The relatedness of the genes and their function was identified using BLASTP (E value < 10) and InterProScan (9). Phylogenetics were conducted using IQ-Tree (10) after MAFFT alignment (11) of the capsid protein (ORF-1). The virus is genetically related to the *Gokushovirinae* subfamily of the *Microviridae* and represents a basal member to the three genera Bdellomicrovirus, Chlamydiamicrovirus, and Spiromicrovirus, as well as multiple other undescribed isolates associated with bacterial endosymbionts of tortoises, marine invertebrates, and insects (Fig. 1).

**TABLE 1:** Similarity and predicted function of the 14 hypothetical ORFs found in *Jodiemicrovirus 1*^*a*^

ORF	Predicted function	Closest hit (accession no.)	Similarity (%)	Coverage (%)	E value
1	Viral capsid	*Microviridae* sp. (AXL15123)	43.06	96	4e−138
2	Transmembrane	—	—	—	—
3	Signal peptide	*Acidimicrobiaceae* (MBB33698)	55.56	84	2.9
4	Unknown	—	—	—	—
5	Transmembrane	—	—	—	—
6	Unknown	—	—	—	—
7	Unknown	—	—	—	—
8	Unknown	—	—	—	—
9	DNA pilot protein	*Microviridae* sp. (YP_009160339)	33.33	40	1e−7
10	Unknown	—	—	—	—
11	Transmembrane	—	—	—	—
12	Signal peptide	—	—	—	—
13	Replication initiator protein	*Microviridae* sp. (AXL15534)	32.00	72	3e−28
14	Unknown	Bacteria (EKD64965)	40.38	41	6.6

a The coding orientation is positive in all cases. Data were analyzed using InterProScan and BLASTP. —, lack of significant protein similarity to any other known sequence data.

Of the 14 hypothetical ORFs, 5 showed similarity to other proteins in GenBank (Table 1). The proteins included a major capsid protein, DNA pilot protein, and the replicator initiator protein, which showed 32 to 44% amino acid similarity to other *Microviridae* (Table 1). One virus was from an environmental sphagnum peat soil sample (12), and two viruses derived from the intestinal tract of Ciona robusta (Tunicata) (marine) (2). Two genes showed closest similarity to hypothetical bacterial genes (Table 1). Seven ORFs were identified internally to other ORFs, indicating the presence of putative overlapping genes recently discovered for the *Microviridae* (13). Based on its relatedness to known *Microviridae*, this genome might represent a novel genus (suggested, *Microviridae*: *Gokushovirinae*: *Jodiemicrovirus*).

To conclude, we present the genome of a bacteriophage likely to infect an undescribed member of the *Anaplasmataceae* which parasitizes the host hepatopancreas, identified via histology, electron microscopy, and genomics (our unpublished data). It may constitute a useful model system for understanding the effect of phage therapy relative to an intracellular bacterium causing disease in crustaceans.

### Data availability.

The complete genome, annotation, and associated forward and reverse reads for this novel virus can be found under accession number MN335165, BioProject number PRJNA574411, and BioSample number SAMN12567204.
